# Prevalence of Suicidality in Major Depressive Disorder: A Systematic Review and Meta-Analysis of Comparative Studies

**DOI:** 10.3389/fpsyt.2021.690130

**Published:** 2021-09-16

**Authors:** Hong Cai, Xiao-Meng Xie, Qinge Zhang, Xiling Cui, Jing-Xia Lin, Kang Sim, Gabor S. Ungvari, Ling Zhang, Yu-Tao Xiang

**Affiliations:** ^1^Unit of Psychiatry, Department of Public Health and Medicinal Administration, Faculty of Health Sciences, Institute of Translational Medicine, University of Macau, Macao, Macao, SAR China; ^2^Centre for Cognitive and Brain Sciences, University of Macau, Macao, Macao, SAR China; ^3^Institute of Advanced Studies in Humanities and Social Sciences, University of Macau, Macao, Macao, SAR China; ^4^The National Clinical Research Center for Mental Disorders & Beijing Key Laboratory of Mental Disorders, Beijing Anding Hospital & the Advanced Innovation Center for Human Brain Protection, Capital Medical University, Macao, Macao, SAR China; ^5^Department of Business Administration, Hong Kong Shue Yan University, Hong Kong, Hong Kong, SAR China; ^6^Department of Rehabilitation Sciences, Hong Kong Polytechnic University, Hong Kong, Hong Kong, SAR China; ^7^West Region, Institute of Mental Health, Buangkok Green Medical Park, Singapore, Singapore; ^8^University of Notre Dame Australia, Fremantle, WA, Australia; ^9^Division of Psychiatry, School of Medicine, University of Western Australia/Graylands Hospital, Perth, WA, Australia

**Keywords:** major depressive disorder, meta-analysis, suicide attempt, comparative study, suicidality

## Abstract

**Background:** Suicidality is common in major depressive disorder (MDD), but there has been no systematic review published about all aspects of suicidality. This meta-analysis and systematic review compared the prevalence of the whole range of suicidality comprising suicidal ideation (SI), suicide plan (SP), suicide attempt (SA), and completed suicide (CS), between patients with MDD and non-MDD controls.

**Methods:** Major international (PubMed, PsycINFO, Web of Science, EMBASE) and Chinese (Chinese Nation Knowledge Infrastructure and WANFANG) databases were systematically and independently searched from their inception until January 12, 2021.

**Results:** Fifteen studies covering 85,768 patients (12,668 in the MDD group and 73,100 in the non-MDD group) were included in the analyses. Compared to non-MDD controls, the odds ratios (ORs) for lifetime, past month, past year, and 2-week prevalence of SI in MDD were 2.88 [95% confidence interval (CI) = 0.30–27.22, *p* = 0.36], 49.88 (95% CI = 2–8.63, *p* < 0.001), 13.97 (95% CI = 12.67–15.41, *p* < 0.001), and 24.81 (95% CI = 15.70–39.22, *p* < 0.001), respectively. Compared to non-MDD controls, the OR for lifetime SP in MDD was 9.51 (95% CI = 7.62–11.88, *p* < 0.001). Compared to non-MDD controls, the ORs of lifetime and past-year prevalence of SA were 3.45 (95% CI = 1.58–7.52, *p* = 0.002), and 7.34 (95% CI = 2.14–25.16, *p* = 0.002), respectively, in MDD patients. No difference in the prevalence of CS between MDD and controls was found (OR = 0.69, 95% CI = 0.23–2.02, *p* = 0.50).

**Conclusions:** MDD patients are at a higher risk of suicidality, compared to non-MDD controls. Routine screening for a range of suicidality should be included in the management of MDD, followed by timely treatment for suicidal patients.

**Systematic Review Registration:** Identifier [INPLASY202120078].

## Introduction

Suicidality is a major global health problem. It is estimated that there are approximately 800,000 people per year who die by suicide, and every 40 seconds, one person completes suicide; in addition, suicide confers huge personal and familial suffering and further compounds healthcare burden (1). For instance, suicide and related problems accounted for 1.4% of the global burden of diseases in 2020 (1).

Suicidality comprises suicidal ideation (SI), suicide plan (SP), suicide attempt (SA), and completed suicide (CS). SI refers to thoughts or wishes about ending one's life, SP refers to making plans for suicide, and SA refers to acts to end one's life (2). Persons with SI, SP, and SA are more likely to have future suicide than those without (3). Therefore, to reduce the risk of future suicide, it is important to understand the patterns of SI, SP, and SA.

Major depressive disorder (MDD) is a common psychiatric disorder, which accounts for up to 87% of CSs (4). Apart from CS, other aspects of suicidality are also common in MDD. For instance, in a recent meta-analysis, the prevalence rates of SI and SA in MDD were 53.1 and 31%, respectively (5, 6).

Studies that compared suicidality between MDD and non-MDD controls yielded conflicting findings. The National Comorbidity Survey in the United States found that the risk of SA in MDD was five-fold higher than in the general population (7). Patients with a major depressive episode have increasing risk of CS after SA (8). A meta-analysis of 20 studies concluded that patients with psychotic depression had a two-fold higher risk of SA compared to their non-psychotic counterparts (9). A thorough search of the literature could not find any meta-analysis comparing the comprehensive range of suicidality (i.e., SI, SP, SA, and CS) between MDD and non-MDD groups. The aim of this meta-analysis was to compare the risk of the whole range of suicidality between those with and without MDD.

## Materials and Methods

### Search Strategy and Selection Criteria

**Figure 1:**
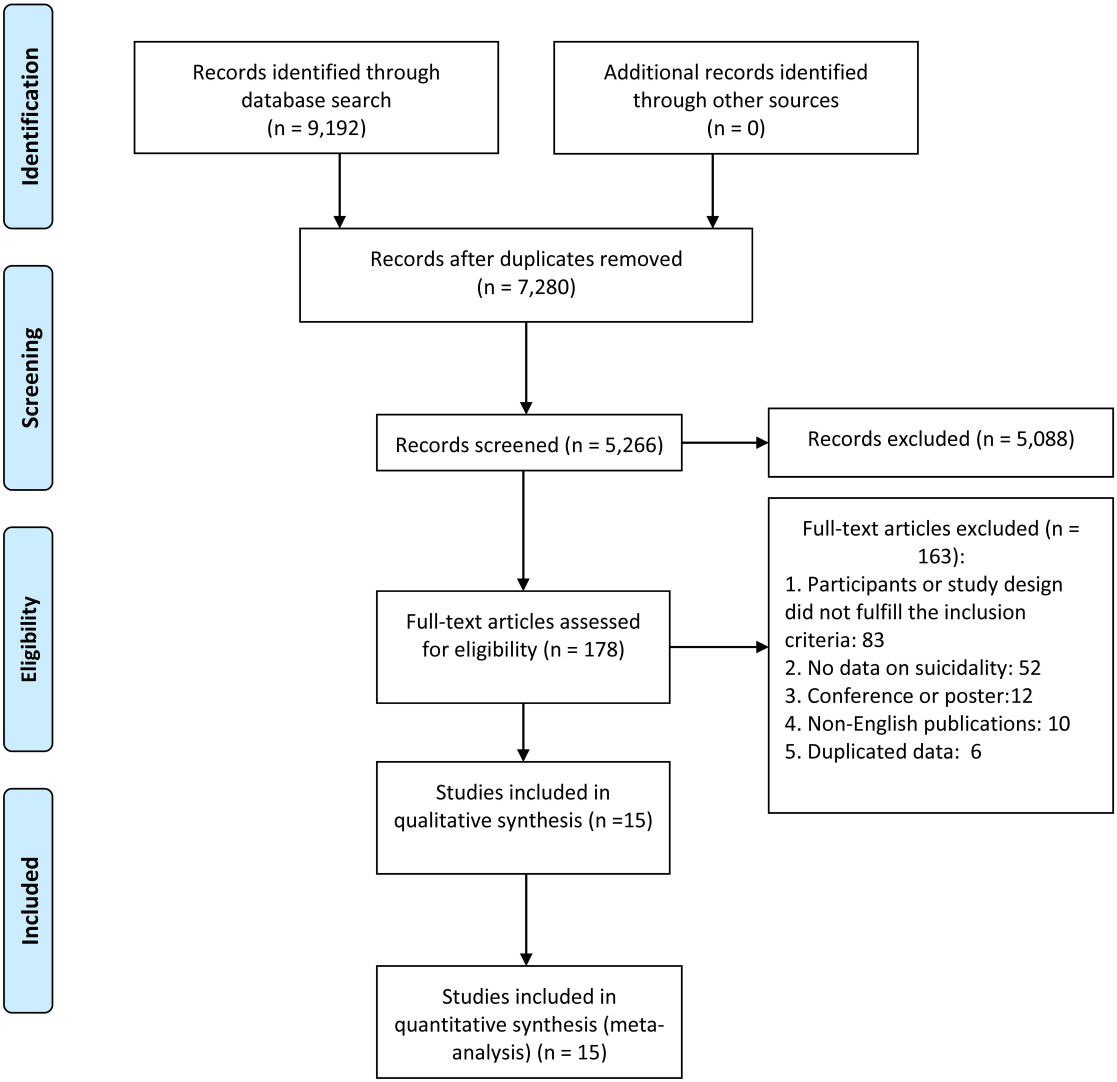
Flow chart of study selection.

This meta-analysis was conducted according to PRISMA (Preferred Reporting Items for Systematic Review and Meta-Analyses) (10). The protocol was registered with INPLASY (International Platform of Registered Systematic Review and Meta-analysis Protocols) (registration number: INPLASY202120078). Two investigators (H.C. and X.M.X.) independently searched the literature in PubMed, PsycINFO, Web of Science, EMBASE, Chinese Nation knowledge Infrastructure (CNKI), and WANFANG databases from their commencement dates until January 12, 2021, using the following search terms: [(suicid^*^ ideation) OR (suicid^*^ idea) OR (suicide thought) OR (suicide plan) OR (self-injurious behavior) OR (self-harm) OR (self-injury) OR (suicid^*^) OR (self-mutilation) OR (self-immolation) OR (self-inflicted) OR (self-slaughter) OR (self-destruction)] AND [(major depress^*^) OR (unipolar depress^*^) OR (Depressive Disorder, Major)] AND [(epidemiology) OR (prevalence) OR (rate)]. The same two investigators independently screened the titles and abstracts and then read the full texts of potentially relevant papers for eligibility. The reference lists of relevant review papers were checked manually to identify missing studies. Uncertainty in the literature search was resolved by a discussion with a senior investigator (X.Y.T.). The process of the literature search is shown in Figure 1.

### Inclusion and Exclusion Criteria

The inclusion criteria according to the PICOS acronym were as follows: participants (P): patients with MDD diagnosed according to international or local diagnostic criteria such as the *Diagnostic and Statistical Manual of Mental Disorders* (11) and the *International Statistical Classification of Diseases and Related Health Problems* (12); intervention (I): not applicable; comparison (C): persons without MDD or other major psychiatric disorders such as schizophrenia or bipolar disorder; outcomes (O): the prevalence of a range of suicidality or data that could generate prevalence of suicidality; and study design (S): case–control or cohort studies (only the baseline data of cohort studies were extracted). Studies involving MDD patients combined with other disorders or other special populations (e.g., children or adolescents, pregnant women, soldiers) were also excluded. If more than one paper was published based on the same dataset, only the one with the largest sample size was included in the analyses.

### Data Extraction and Study Quality Assessment

The same two investigators independently conducted the data extraction by using a standard form. Study and patient characteristics, such as the first author, year of publication, survey time, study location and design, source of patients (e.g., inpatients, outpatients, community, or mixed), total sample size, diagnostic criteria of MDD and MDD sample size, non-MDD group diagnoses and sample size, proportion of males, type of suicidality, mean age, and timeframe of suicidality, were extracted. Study quality was assessed using a standardized instrument for epidemiological studies (13, 14) with the following eight items: (1) target population was defined clearly; (2) probability sampling or entire population surveyed; (3) response rate was ≥80%; (4) non-responders were clearly described; (5) sample was representative of the target population; (6) data collection methods were standardized; (7) validated criteria were used to diagnose MDD; and (8) prevalence estimates were given with confidence intervals (CIs) and detailed by subgroups (if applicable). The total score ranges from 0 to 8. Studies with a total score of “7–8” were considered as “high quality,” “4–6” as “moderate quality,” and “0–3” as “low quality” (15).

### Statistical Analysis

The meta-analysis was conducted using the Comprehensive Meta-Analysis, version 2.0 (Biostat Inc., Englewood, NJ, USA). The random-effects model was used to calculate the pooled prevalence of suicidality and their 95% confidence intervals (95% CIs) due to the different sampling methods and patients' demographic characteristics between studies. Heterogeneity between studies was assessed with the *I*^2^ statistic; *I*^2^ > 50% indicated high heterogeneity. Sensitivity analyses were performed to identify outlying studies by excluding studies one by one. Publication bias was estimated with funnel plots and the Egger test. A *p* < 0.05 was considered as statistically significant (two-tailed).

## Results

### Characteristics of the Included Studies

One hundred seventy-eight full-text articles of the 9,192 studies initially identified in the literature search were assessed for eligibility. Fifteen studies fulfilled the entry criteria and were included in the meta-analysis (Figure 1). Twelve studies assessed SA, five studies assessed SI, one study assessed SP, one study assessed unspecified suicidality (i.e., the subtype of suicidality was not specified), and two studies targeted only CS. The sample size ranged from 47 to 42,551; the mean ages ranged from 30.8 to 44.5 years (Table 1). Twelve studies were cross-sectional. Study quality assessment scores ranged from 3 to 7; 1 study was of low, 13 studies were of moderate, and 2 studies were of high quality (Supplementary Table 1).

**Table 1:** Characteristics of the studies included in the meta-analysis.

								**MDD group**		**Control group**							
**No**.	**First author**	**Reference**	**Publication year**	**Study location**	**Survey time**	**Mean age (years)**	**Total (n)**	**Diagnose**	**MDD (n)**	**Diagnose**	**Control (n)**	**Study design**	**Men (%)**	**Source of patients**	**Type of suicidality**	**Timeframe of suicidality**	**Quality assessment**
1	Li	(17)	1996	China	1988–1994	35.2	52	CCMD-2	30	Neurosis	22	Case–Control	44.2	Inpatients	SA	NA	4
2	Li	(20)	2006	China	NA	49.93	5,802	*DSM-IV*	198	Non-MDD	5,584	Cross-Sectional	48.3	Mixed	SA	Lifetime, last time	6
3	Soderholm	(21)	2020	Finland	NA	30.8	81	*DSM-IV*	50	BPD	31	Cohort	35.8	Outpatient	SA, SI, SB	Lifetime, recent	6
4	Salloum	(19)	1995	USA	NA	NA	3,175	*DSM-III*	2,421	AUD	754	Cross-Sectional	40.1	Mixed	SA	NA	4
5	Choi	(22)	2019	Korea	2006−2007	44.5	12,324	*DSM-IV*	753	Non-MDD	11,571	Cross-Sectional	39.2	Mixed	SA, SP, SI	Lifetime	7
6	Moffitt	(18)	2007	New Zealand	NA	NA	425	*DSM-IV*	212	Non-MDD	213	Retro- spective	48.7	Community	SA	NA	6
7	Holmstrand	(23)	2008	Sweden	NA	43.4	116	*DSM-III*	81	Dysthymia	35	Cross-Sectional	36.2	Mixed	SA, CS	Lifetime	4
8	Chen	(24)	1996	USA	NA	NA	6,498	*DSM-III*	801	Non-MDD	5,697	Cross-Sectional	43.5	NA	SA	Lifetime	4
9	Li	(25)	2017	China	2016.3–2016.6	38.1	5,189	*DSM-IV*	190	Non-MDD	4,999	Cross-Sectional	33.8	Outpatient	SI	Past month	6
10	Goldney	(26)	2002	Australia	NA	NA	3,010	*DSM-IV*	205	Non-MDD	2,805	Cross-Sectional	NA	Mixed	SI	Last 2 weeks	4
11	Sagud	(27)	2020	Croatia	NA	NA	371	*DSM-IV*	178	Non- psychiatric	193	Cross-Sectional	32.9	NA	SA	Lifetime	4
12	Ma	(28)	2009	China	2003.4	NA	4,767	CIDI	153	Non-MDD	4,614	Cross-Sectional	45.9	NA	SA	Lifetime	6
13	Bronisch	(16)	1994	Germany	1974–1982	NA	360	*DSM-III*	54	Non-MDD	316	Cohort	NA	Inpatients	SA	NA	5
14	Areen	(29)	2021	USA	2018	NA	42,551	*DSM-5*	6,999	Non-MDD	35,552	Cross-Sectional	NA	Community	SA, SI	Past 1 year	7
15	Axelsson	(30)	1992	Sweden	NA	NA	47	*DSM-III*	33	Paranoid disorder	14	Cross-Sectional	1	Mixed	CS	Lifetime	3

*MDD, major depressive disorder; BPD, borderline personality disorder; DSM, Diagnostic and Statistical Manual of Mental Disorders; CIDI, Composite Interview Diagnostic Instrument; AUD, alcohol use disorder; NA, not available; CCMD-2, Chinese Classification of Mental Disorders*.

### Suicidal Ideation

**Figure 2:**
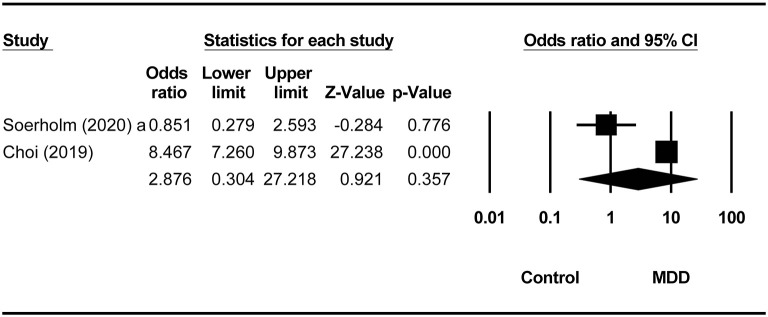
Comparison of lifetime prevalence of suicide ideation between the MDD group and non-MDD groups.

Compared to non-MDD controls, the odds ratios (ORs) for lifetime, past month, past year, and 2-week prevalence of SI in MDD were 2.88 (95% CI = 0.30–27.22, *p* = 0.36, *I*^2^ = 93.77%), 49.88 (95% CI = 2–8.63, *p* < 0.001, *I*^2^ = 0), 13.97 (95% CI = 12.67–15.41, *p* < 0.001, *I*^2^ = 0), and 24.81 (95% CI = 15.70–39.22, *p* < 0.001, *I*^2^ = 0), respectively (Figure 2). Compared to borderline personality disorder, no significant increase of SI was found in the MDD group (OR = 1.47, 95% CI = 0.60–3.63, *p* = 0.40, *I*^2^ = 0) (Table 2).

**Table 2:** Summary of overall prevalence of suicide behaviors between the MDD group and non-MDD groups.

		**MDD group**		**Non-MDD group**					
	**Categories (no. of studies)**	**Events**	**Total**	**Events**	**Total**	**OR**	**95% CI (%)**	***p* value**	***I* ^**2**^**
**Suicide attempts**									
Lifetime	7	379	2,214	666	27,725	3.45	(1.58–7.52)	0.002	94.81
Past year	2	207	7,197	265	41,552	7.34	(2.14–25.16)	0.002	88.64
Recent	1	3	50	5	31	0.33	(0.07–1.50)	0.15	0
**Suicide ideation**									
Lifetime	2	451	803	1,470	11,602	2.88	(0.30–27.22)	0.36	93.77
Recent	1	29	50	15	31	1.47	(0.60–3.63)	0.40	0
Past month	1	67	190	54	4,999	49.88	(33.42–74.46)	<0.001	0
Past year	1	1,409	6,999	630	35,552	13.97	(12.67–15.41)	<0.001	0
Past 2 weeks	1	50	205	36	2,805	24.81	(15.70–39.22)	<0.001	0
**Suicide plan**									
Lifetime	1	136	753	262	11,571	9.51	(7.62–11.88)	<0.001	0
**Suicide behavior**									
Lifetime	1	12	50	20	31	0.17	(0.06–0.46)	<0.001	0
Recent	1	8	50	8	31	0.55	(0.18–1.65)	0.29	0
**Completed suicide**									
Lifetime	2	10	114	6	49	0.69	(0.23–2.02)	0.50	0

### Suicide Attempt

**Figure 3:**
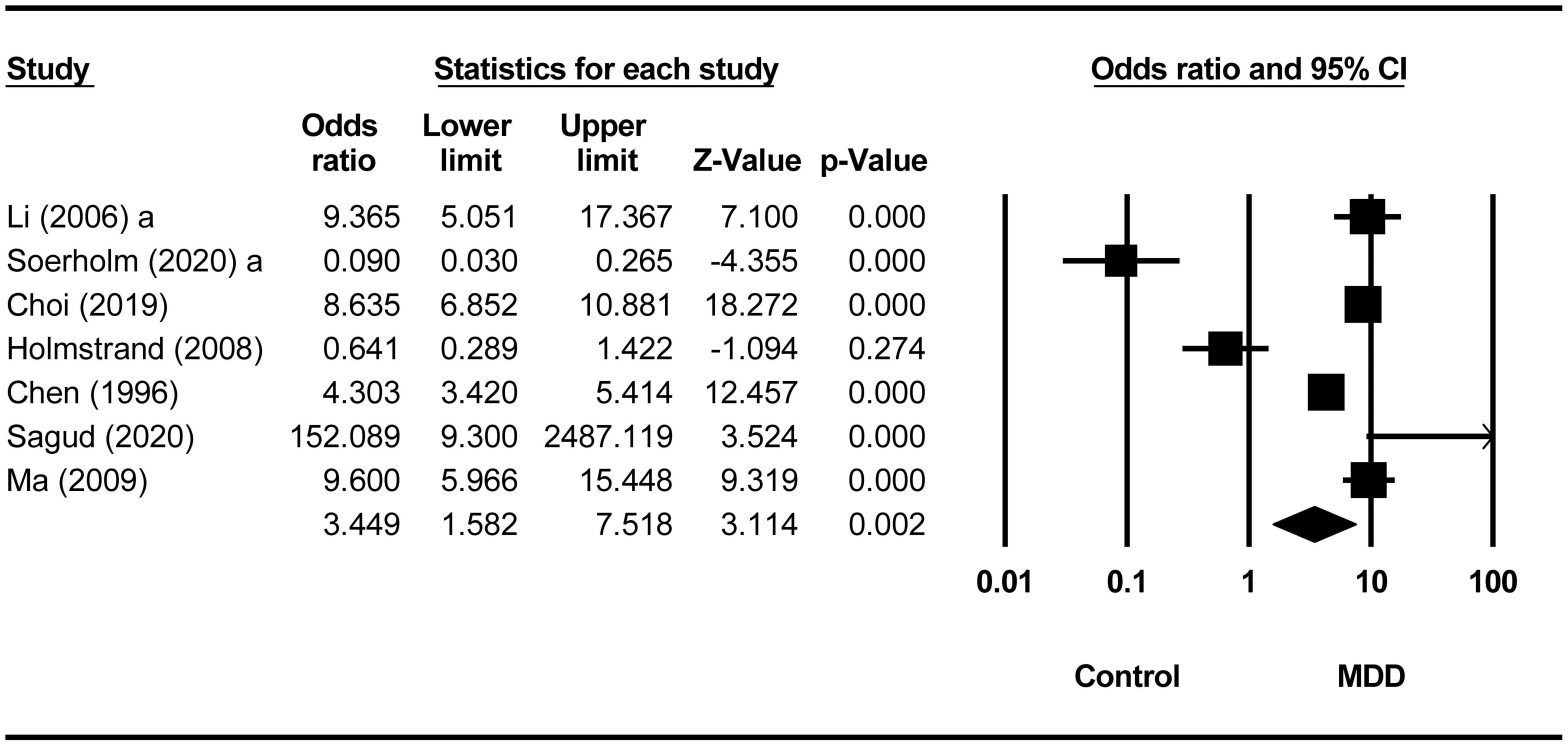
Comparison of lifetime prevalence of suicide attempts between MDD and non-MDD groups.

**Figure 4:**
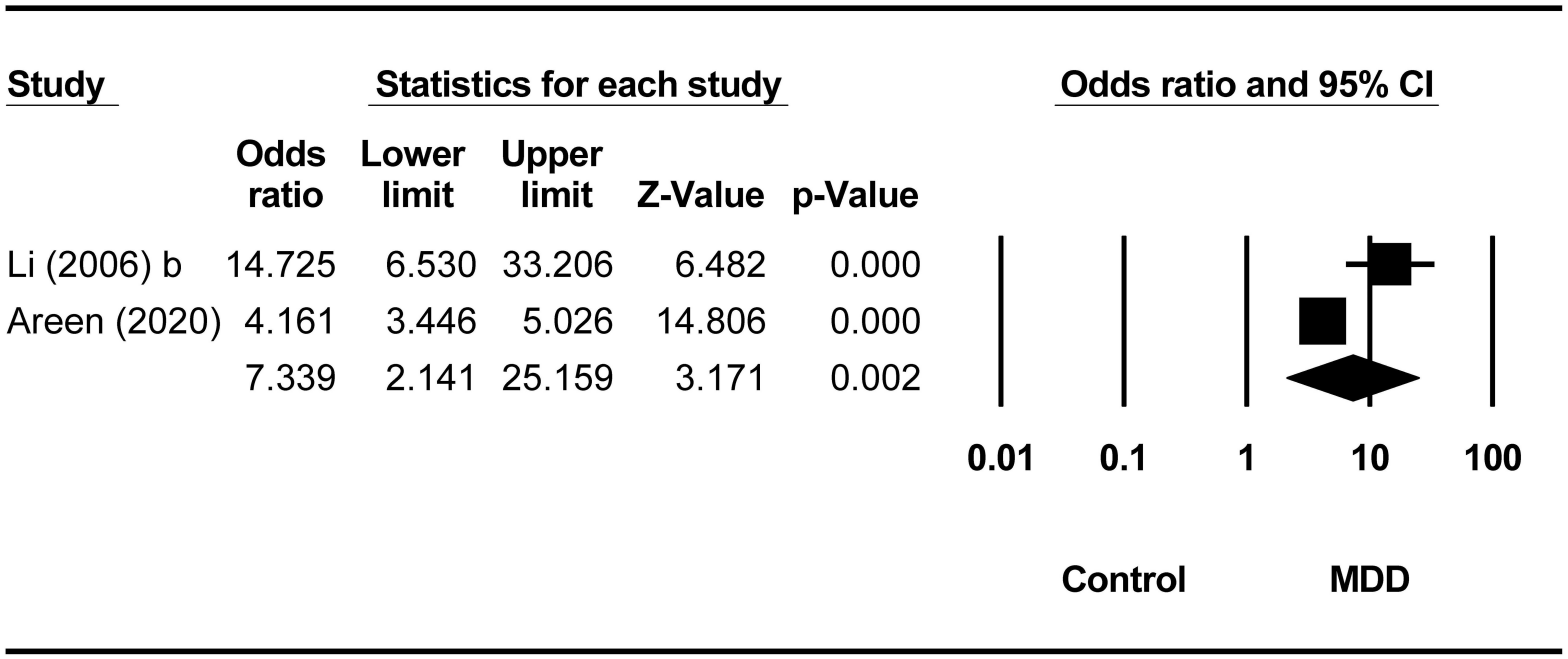
Comparison of 1-year prevalence of suicide attempts between the MDD and non-MDD groups.

Compared to non-MDD controls, the pooled ORs for lifetime and past-year SA in the MDD group were 3.45 (95% CI = 1.58–7.52, *p* = 0.002, *I*^2^ = 94.81%; Figure 3) and 7.34 (95% CI = 2.14–25.16, *p* = 0.002, *I*^2^ = 88.64%), respectively (Figure 4). There was no difference in the prevalence of recent SA between MDD and borderline personality disorder (OR = 0.33, 95% CI = 0.07–1.50, *p* = 0.15, *I*^2^ = 0). Four studies (16–19) did not report the timeframe of SA (Table 1). In the study by Salloum et al. (19), the prevalence of SA was 29.7% (719/2,421) in MDD, whereas the corresponding figure was 21.8% (164/754) in alcohol use disorder (OR = 1.52, 95% CI = 1.25–1.85, *p* < 0.001). In the study by Li and Wang (17), the prevalence of SA was 33.3 % (10/30) in MDD, and it was 0 (0/20) in neurotic disorders (OR = 23.05, 95% CI = 1.27–418.67, *p* = 0.034). In the study by Moffitt et al. (18), the prevalence of SA was 3.7% (8/212) in MDD, whereas the corresponding figure was 0 (0/213) in the general population (OR = 17.75, 95% CI = 1.02–309.48, *p* = 0.049). Bronisch and Wittchen (16) found the prevalence of SA was 14.8% (8/54) in MDD and 1.9% (6/316) in the general population (OR = 8.99, 95% CI = 2.98–27.07, *p* < 0.001) (Table 2).

### SP and CS

Compared to non-MDD controls, the OR for lifetime SP in MDD was 9.51 (95% CI = 7.62–11.88, *p* < 0.001, *I*^2^ = 0). No difference in the prevalence of CS between MDD and controls was found (OR = 0.69, 95% CI = 0.23–2.02, *p* = 0.50, *I*^2^ = 0). Two studies reported the lifetime CS (Table 1). Holmstrand et al. (23) reported the prevalence of CS was 9.9% (8/81) in MDD, and 14.3% in dysthymia (OR = 0.66, 95% CI = 0.20–2.17, *p* = 0.49). Axelsson and Lagerkvist-Briggs (30) found the prevalence of CS was 6.1 (2/33) in MDD and 7.1% (1/14) in delusional disorder (OR = 0.84, 95% CI = 0.07–10.08, *p* = 0.89). Because of the small number of included studies, subgroup analysis and metaregression analyses could not be performed (Table 2).

### Unspecified Suicidality

Compared to borderline personality disorder, MDD had a lower risk of lifetime suicidality (OR = 0.17, 95% CI = 0.06–0.46, *p* < 0.001, *I*^2^ = 0), whereas no difference in the prevalence of recent suicidality between borderline personality disorder and MDD groups was found (OR = 0.55, 95% CI = 0.18–1.65, *p* = 0.29, *I*^2^ = 0) (Table 2).

### Publication Bias and Sensitivity Analysis

The funnel plot of lifetime prevalence of SA did not show publication bias (Egger test: *t* = −2.37, *p* = 0.47; Supplementary Figure 1). After removing each study from studies reporting lifetime SA, no outlying study that could have significantly changed the primary results was found.

## Discussion

To the best of our knowledge, this was the first meta-analysis and systematic review that compared the comprehensive range of suicidality between MDD and other psychiatric disorders and a healthy control group. Compared to non-MDD controls, MDD patients had a significantly higher risk of lifetime and past-year SA than non-MDD controls, which is consistent with previous findings that focused on psychotic MDD (9, 31). Non-MDD controls in this meta-analysis belonged to diverse diagnostic groups including borderline personality disorder, dysthymia, delusional disorder, and healthy persons.

The increased suicidality in MDD could be due to several reasons. Symptoms in MDD, such as feelings of hopelessness, worthlessness, delusionally depressive thoughts, anxiety, and sleep disturbances, directly and indirectly increase the risk of SA (32, 33). In addition, psychosocial factors associated with MDD, such as disruption of marital and family connections, could also increase the risk of suicidality (29, 33).

Suicidality lies on a continuum ranging from SI, SP, SA, to CS (34). Apart from sociocultural factors, the increased risk of suicidality in MDD could be associated with biological factors, such as the uncoupled N-acetylaspartate–glutamatergic metabolism in the anterior cingulate cortex (35) and the impaired executive functions and impulsivity control caused by decreased structural connectivity in the frontosubcortical circuit (36). Because of the limited number of studies on CS in MDD, the prevalence estimates of CS were not synthesized in this meta-analysis. Major risk factors for suicidality, particularly future CS in MDD, included severe depressive and psychotic symptoms (37, 38) and treatment resistance (39). The roles of these factors, however, were not explored in this meta-analysis owing to insufficient data in the included studies.

In this meta-analysis, the lifetime prevalence of SA was higher in borderline personality disorder than in MDD, which could be explained by the heightened sensitivity to abandonment, feelings of emptiness, and outbursts of anger, which are features of borderline personality disorder (40). Dysthymia also raises the frequency of suicidality (38, 41). In this systematic review, no significant differences in the prevalence of SA and CS between dysthymia and MDD were found, which is similar to previous findings (38, 41). Alcohol use disorder also has increased risk of suicidality (42). In this meta-analysis, the prevalence of SA in MDD was significantly higher than in alcohol use disorder.

Mental health professionals should integrate suicide prevention measures into clinical practice and devise effective communication channels designed to prevent suicide by changing knowledge, attitudes, and behaviors of MDD patients (33, 43–45). It is imperative to identify risk factors of suicidality in MDD, especially those that could accelerate the transition from SI and SP, to SA and to CS. It is also important to conduct regular screening targeting suicidality and risk factors in MDD (45).

Several limitations of the study should be noted. First, similar to many meta-analyses of comparative studies (9, 46, 47), a relatively high level of heterogeneity was encountered. Heterogeneity could be partly due to various types of controls, patient demographic characteristics, sampling methods, and measures on suicidality. Second, because of the small number of studies with each type of controls, subgroup analyses could not be performed to examine their moderating effect on the results. For the same reason, subgroup and metaregression analyses for each type of suicidality could not be performed. Third, most included studies had a case–control design; therefore, the possibility of recall bias about suicidality could not be excluded. Fourth, several moderators relevant to the prevalence of suicidality in MDD, such as age, gender, general health status, and social circumstances, could not be examined because of insufficient data.

In conclusion, MDD patients are at a higher risk of suicidality compared to diagnostically heterogeneous non-MDD controls. Considering the enormous suffering for patients and their relatives related to suicidality, as well as the negative impact of suicidality on health outcomes, regular screening for the whole range of suicidality should be included in clinical evaluation and management of MDD, and timely treatment should be provided for suicidal patients.

## Data Availability Statement

The original contributions presented in the study are included in the article/Supplementary Material, further inquiries can be directed to the corresponding author/s.

## Author Contributions

Y-TX and LZ: study design. HC, X-MX, QZ, XC, and J-XL: collection, analyses, and interpretation of dat. HC, GU, and Y-TX: drafting of the manuscript. KS: critical revision of the manuscript. All authors approval of the final version for publication.

## Funding

The study was supported by the National Science and Technology Major Project for investigational new drugs (2018ZX09201-014), the Beijing Municipal Science & Technology Commission (Grant No. Z181100001518005), and the University of Macau (MYRG2019-00066-FHS).

## Conflict of Interest

The authors declare that the research was conducted in the absence of any commercial or financial relationships that could be construed as a potential conflict of interest.

## Publisher's Note

All claims expressed in this article are solely those of the authors and do not necessarily represent those of their affiliated organizations, or those of the publisher, the editors and the reviewers. Any product that may be evaluated in this article, or claim that may be made by its manufacturer, is not guaranteed or endorsed by the publisher.
