# Supplementary material for: Prevalence of Suicidality in Major Depressive Disorder: A Systematic Review and Meta-Analysis of Comparative Studies
Source: Front Psychiatry. 2021 Sep 16;12:690130. doi: 10.3389/fpsyt.2021.690130 (PMC8481605; doi:10.3389/fpsyt.2021.690130)
Supplement: Supplementary file 1 [file Table_1.DOC]

Supplementary Table 1. Quality assessment of included studies

|  | **1. Target population is clearly defined** | **2. Probability sampling OR entire population surveyed** | **3. Is the response rate ≥80%?** | **4. Are non-responders clearly described?** | **5. Is the sample representative of the target population?** | **6. Were data collection methods standardized?** | **7. Were validated criteria used to diagnose MDD?** | **8. Are the prevalence estimates given with confidence intervals and detailed by subgroups**  **(if applicable)?** | **Total score** |
| --- | --- | --- | --- | --- | --- | --- | --- | --- | --- |
| Li (Xuanpei Li, 1996) | 1 | 0 | 0 | 0 | 1 | 1 | 1 | 0 | 4 |
| Li (Xianyun Li, 2006) | 1 | 1 | 0 | 1 | 1 | 1 | 1 | 0 | 6 |
| Söderholm (Soderholm et al., 2020) | 1 | 1 | 0 | 1 | 1 | 1 | 1 | 0 | 6 |
| Salloum (Salloum et al., 1995) | 1 | 0 | 0 | 0 | 1 | 1 | 1 | 0 | 4 |
| Choi (Choi et al., 2019) | 1 | 1 | 1 | 1 | 1 | 1 | 1 | 0 | 7 |
| Moffitt (Moffitt et al., 2007) | 1 | 1 | 1 | 0 | 1 | 1 | 1 | 0 | 6 |
| Holmstrand (Holmstrand et al., 2008) | 1 | 0 | 0 | 0 | 1 | 1 | 1 | 0 | 4 |
| Chen (Chen & Dilsaver, 1996) | 1 | 0 | 0 | 0 | 1 | 1 | 1 | 0 | 4 |
| Li (Li et al., 2017) | 1 | 0 | 1 | 1 | 1 | 1 | 1 | 0 | 6 |
| Goldney (Goldney et al., 2002) | 1 | 0 | 0 | 0 | 1 | 1 | 1 | 0 | 4 |
| Sagud (Sagud et al., 2020) | 1 | 0 | 0 | 0 | 1 | 1 | 1 | 0 | 4 |
| Ma (Ma et al., 2009) | 1 | 1 | 1 | 1 | 1 | 1 | 1 | 0 | 7 |
| Bronisch (Bronisch & Wittchen, 1994) | 1 | 0 | 0 | 1 | 1 | 1 | 1 | 0 | 5 |
| Areen (Omary, 2020) | 1 | 1 | 1 | 1 | 1 | 1 | 1 | 0 | 7 |
| Axelsson (Axelsson & Lagerkvist-Briggs, 1992) | 1 | 0 | 0 | 0 | 1 | 1 | 1 | 0 | 4 |

Supplementary Figure 1. Publication bias of the included studies reporting lifetime prevalence of SA in MDD compared with non-MDD groups. (Plotted by random effects)


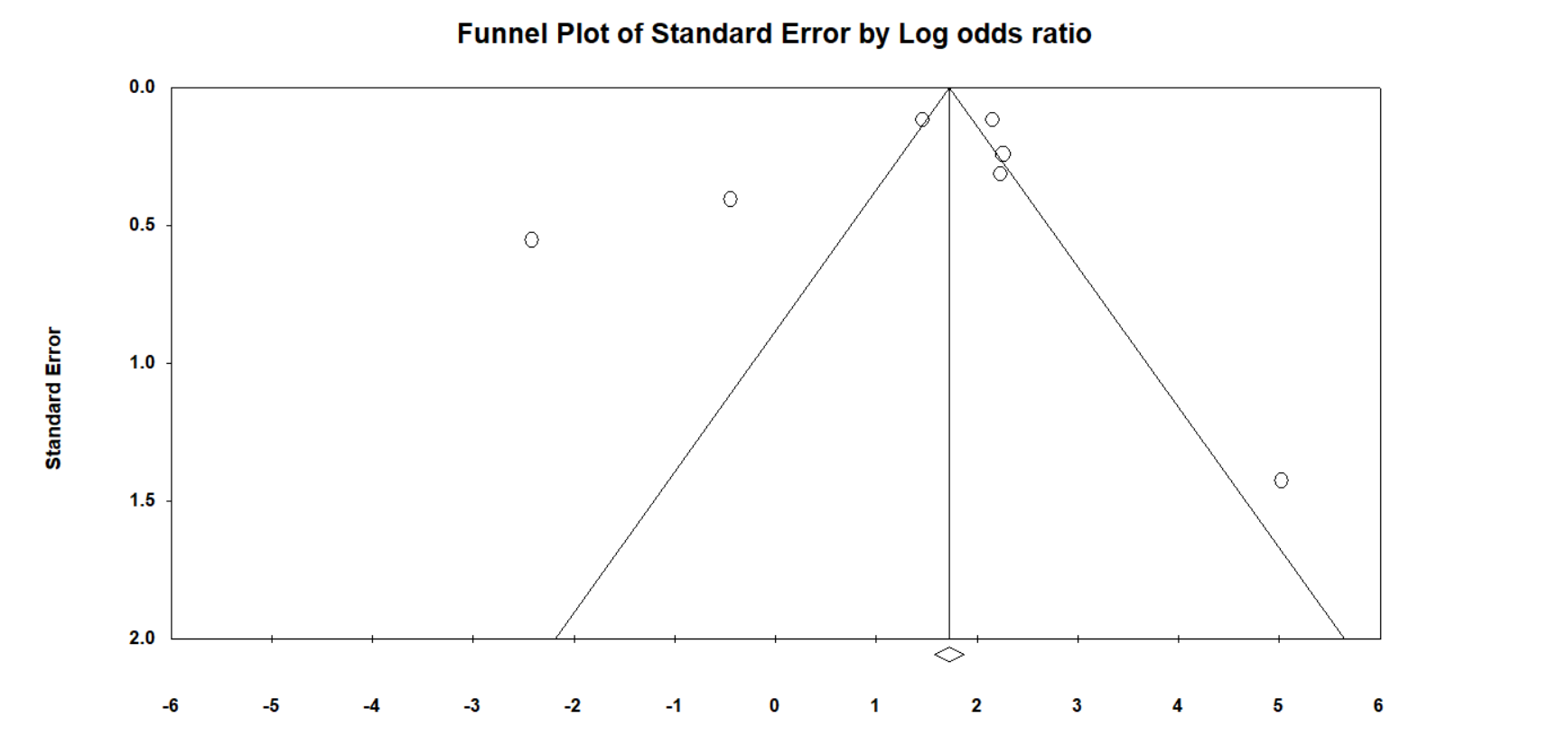


Egger’s test: t = -2.37, p=0.47
